# Choice reaching with a LEGO arm robot (CoRLEGO): The motor system guides visual attention to movement-relevant information

**DOI:** 10.1016/j.neunet.2015.10.005

**Published:** 2015-12

**Authors:** Soeren Strauss, Philip J.W. Woodgate, Saber A. Sami, Dietmar Heinke

**Affiliations:** a Centre for Computational Neuroscience and Cognitive Robotics, School of Psychology, University of Birmingham, Birmingham B15 2TT, United Kingdom

**Keywords:** Target reaching, Visual attention, Transcranial direct current stimulation (tDCS), Motor cortex, Neurobiologically inspired robotics, LEGO Mindstorms

## Abstract

We present an extension of a neurobiologically inspired robotics model, termed CoRLEGO (Choice reaching with a LEGO arm robot). CoRLEGO models experimental evidence from choice reaching tasks (CRT). In a CRT participants are asked to rapidly reach and touch an item presented on the screen. These experiments show that non-target items can divert the reaching movement away from the ideal trajectory to the target item. This is seen as evidence attentional selection of reaching targets can leak into the motor system. Using competitive target selection and topological representations of motor parameters (dynamic neural fields) CoRLEGO is able to mimic this leakage effect. Furthermore if the reaching target is determined by its colour oddity (i.e. a green square among red squares or vice versa), the reaching trajectories become straighter with repetitions of the target colour (colour streaks). This colour priming effect can also be modelled with CoRLEGO. The paper also presents an extension of CoRLEGO. This extension mimics findings that transcranial direct current stimulation (tDCS) over the motor cortex modulates the colour priming effect (Woodgate et al., 2015). The results with the new CoRLEGO suggest that feedback connections from the motor system to the brain’s attentional system (parietal cortex) guide visual attention to extract movement-relevant information (i.e. colour) from visual stimuli. This paper adds to growing evidence that there is a close interaction between the motor system and the attention system. This evidence contradicts the traditional conceptualization of the motor system as the endpoint of a serial chain of processing stages. At the end of the paper we discuss CoRLEGO’s predictions and also lessons for neurobiologically inspired robotics emerging from this work.

## 1 Introduction

Traditionally the motor system is seen as the endpoint of a serial chain of processing stages. This framework assumes that the motor system ‘simply’ reads out previous processing in order to execute a movement (e.g. Marr, 1982). However recent research has shown that the motor system and other processing stages are more closely linked. For instance, a series of studies showed that cognitive processes (e.g. language processing; Spivey, Grosjean, & Knoblich, 2005; numerical representation; Song & Nakayama, 2008a) can leak into reaching movements (see Song & Nakayama, 2009 for a review). This evidence for parallel feed forward processing is also complemented by evidence suggesting feedback influences. For instance, learning processes in the motor system can change the psychophysical judgment of perceptual stimuli (e.g. Ostry, Darainy, Mattar, Wong, & Gribble, 2010), and visual discrimination is better at locations that form movement targets than elsewhere (e.g. Deubel, Schneider, & Paprotta, 1998). The present paper focuses on the interplay of the motor system with visual selective attention.

Evidence for the interactions between the motor system and visual selective attention comes from studies by Song and Nakayama (Song and Nakayama, 2006, Song and Nakayama, 2008b; see Song & Nakayama, 2009 for a review) and Woodgate, Strauss, Sami, and Heinke (2015) and will be summarized in the next section. In Section  3 we will present a neurobiologically inspired robotics model of this evidence. The model is called CoRLEGO (Choice reaching with a LEGO robot arm) and was first published by Strauss and Heinke (2012). Section  4 will present an extension of CoRLEGO to accommodate novel findings from Woodgate et al. (2015). Section  4 will discuss the implications of our work. Section  5 will summarize lessons for neurobiologically inspired robotics from our work.

### 1.1 Selective attention and reaching movements

Visual scenes are highly complex. Selective attention is generally assumed to deal with this complexity by filtering out information irrelevant to the task at hand. The well-known biased competition theory (e.g. Desimone & Duncan, 1995) postulates that filtering of irrelevant items is implemented as a competition process in the brain. The various objects in scenes are assumed to compete for the observer’s behavioural response. Competition can be biased by a wide range of factors, such as the physical properties of objects, the knowledge of the observer, the repetition of target features, etc. A commonly used experimental method to examine these factors is the visual search task (see Eckstein, 2011 and Müller & Krummenacher, 2006 for reviews). Typically in this task participants are asked to indicate the absence or presence of a pre-defined target object among non-target objects (distractors) by pressing a specific key on a keyboard. Within the biased competition framework the time it takes participants to press the key (reaction times) is assumed to be an index for the strength of the competition between items on the screen. A particularly well-known finding relevant to the present paper is that if the target items differ by one feature (e.g. a red square among green squares) there is relatively little competition resulting in short reaction times; also termed the pop-out effect (e.g. Treisman & Gelade, 1980). Nevertheless, studies by Maljkovic and Nakayama (1994) indicate that the repetition of target features (e.g. streaks of the same colour) can result in faster resolution of inter-item competition, i.e. the classic priming of pop-out (POP) effect. Note that in these tasks participants had to discriminate a feature of the target item rather than making a present/absent response. Target discrimination turned out to be a crucial pre-condition for the POP-effect which was not found in the present/absent task.

The choice reaching task (CRT) of Song and Nakayama combines the visual search task with reaching movements. In a CRT participants are asked to rapidly reach and touch an item presented on the screen. Song and Nakayama (2008b) employed two types of display. One type consisted of three squares and the movement target was indicated by its colour-oddity (e.g. a red square among green squares, or vice versa). The other display type contained just one item (either a green or red square). The odd-colour display can be considered to be a harder search task than the single target display. Song and Nakayama (2008b) found that search difficulty was reflected in two measures: the initiation latency of movement (IL) and the maximum deviation of the reach trajectory (MD) from the ideal path (straight line). When the task was easy IL was short and MD was small while when the task was hard IL was long and MD was larger. Moreover and consistent with the biased competition theory, Song and Nakayama interpreted the MD-effect as reflecting the competition between items on the screen during the selection process which ‘leaks’ into motor control (see also Song & Nakayama, 2009). Participants may begin their movements before selection is completed (especially in the mixed design) and, as a consequence, the non-target items (distractors) can divert the reaching movement away from the ideal trajectory to the target item. In another study, Song and Nakayama (2006) showed that when the target colour is repeated from trial-to-trial both latency and deviation are reduced. Hence both measurements index the POP-effect discussed earlier.

In Woodgate et al. (2015) we applied transcranial direct current stimulation (tDCS) to the motor cortex while participants completed a CRT. tDCS is an electrical neurostimulation technique that passes constant ultra-low currents to brain regions via a pair of electrodes placed on the scalp. Anodal stimulation refers to when the positively charged electrode (anode) is placed over the stimulated brain area whilst cathodal stimulation uses negatively charged current. tDCS is known to increase (anodal tDCS) or decrease (cathodal tDCS) excitability of the underlying cortex (e.g. Nitsche & Paulus, 2001). In both cases, there are measurable behavioural consequences (e.g. Reis et al., 2009). It is worth noting that on the face of it electrical stimulation on the scalp seems fairly non-specific. However a recent study combining tDCS over the motor cortex with electroencephalography (EEG) demonstrated effects predominantly on the motor cortex and functionally-related areas (Notturno, Marzetti, Pizzella, Uncini, & Zappasodi, 2014)

In fact, Woodgate et al. (2015) also stimulated participants over the motor cortex while they performed CRTs. They found that when the experimental design of the CRT was similar to Song and Nakayama (2008b) (i.e. randomly intermixed three item vs. single items trials with no target colour streaks) tDCS showed little influence on the participants’ reaching behaviour. However, when the experiment contained streaks of target colour (similar to Song & Nakayama, 2006), tDCS affected the reaching trajectories. Woodgate et al. (2015) concluded from these findings that the motor system may be involved in the guidance of attention when target properties are predictable. Such an interpretation is consistent with findings referred to in the introduction that the motor system can influence perceptual processing (e.g. Deubel et al., 1998 and Ostry et al., 2010). However, there are many intriguing aspects of Woodgate’s et al. (2015) findings. We will discuss them in more detail in Section  3 in the context of the extension of CoRLEGO.

## 2 CoRLEGO

CoRLEGO is based on Song and Nakayama’s (2009) theory that the target selection process leaks into the motor stage (Strauss & Heinke, 2012). For the implementation of the target selection stage CoRLEGO draws on our laboratory’s competition models of attention (e.g. [Bibr br000050][Bibr br000075] and Zhao, Humphreys, & Heinke, 2012). The motor stage utilizes Erlhagen and Schoener’s (2002) dynamic neural field (DNF) theory that postulates that movement parameters are encoded in a topological representation. In such a map (field), similar parameter values are encoded in a spatial neighbourhood whereas very different values are represented at locations that are far apart in the neural field. The output activation of the neural field indicates how likely it is that a particular parameter value influences the movement. In addition (Erlhagen & Schoener, 2002) assumes that the behaviour of dynamic neural fields is governed by local excitatory and global inhibitory connections (see Eq. (A.1)).

However, as the model is part of a real world setting inevitably not all mechanisms can be considered a true model of human processes and instead have to be considered as technical solutions. Despite this, CoRLEGO still operationalises central aspects of Song and Nakayama’s (2009) theory. In the following we will first summarize the technical aspects of CoRLEGO and refer the reader to (Strauss & Heinke, 2012) for details. Subsequently we will give an overview of the model CoRLEGO is based on and summarize the results published by Strauss and Heinke (2012). Strauss and Heinke (2012) did not discuss how CoRLEGO relates to brain areas. We will remedy this shortcoming at the end of this section.

### 2.1 Setup and technical solutions

**Fig. 1:**
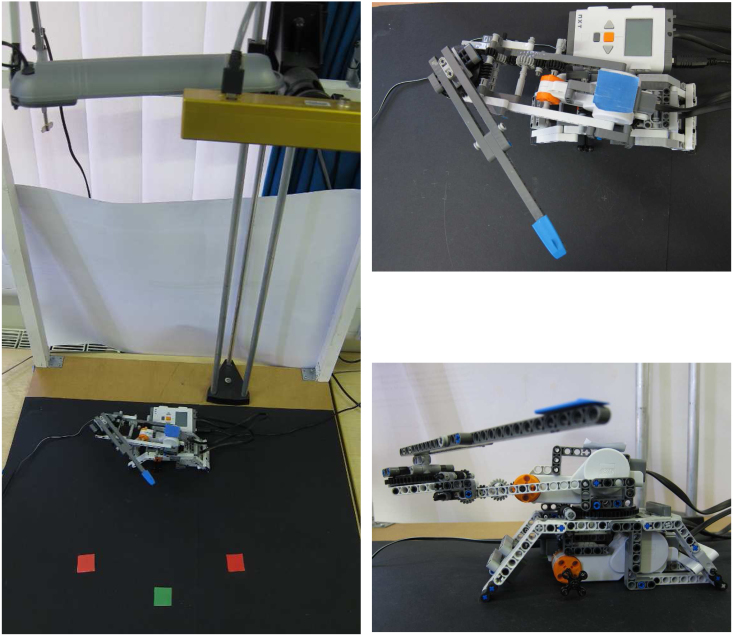
Set-up of CoRLEGO with details of the arm. (For interpretation of the references to colour in this figure legend, the reader is referred to the web version of this article.)

Fig. 1 shows the set-up. As the crucial effects of the CRT can be captured by two-dimensional movements (i.e. diversion from a straight line), we designed a planar LEGO robot arm with two joints. The robot arm was built with LEGO Mindstorms NXT kits. The total length of the arm is approximately 36 cm long (forearm 19 cm and upper arm 17 cm). The robot arm and its environment are filmed with a camera from a birds-eye view (see yellow box in top right corner of Fig. 1). The distance between camera and table is 90 cm. The birds-eye view constitutes a strong simplification from the real experimental set-up. However the exact viewing angle can be considered not to be crucial in the CRT-task. It also simplifies the necessary coordinate transformations from target coordinates to joint movements.

**Fig. 2:**
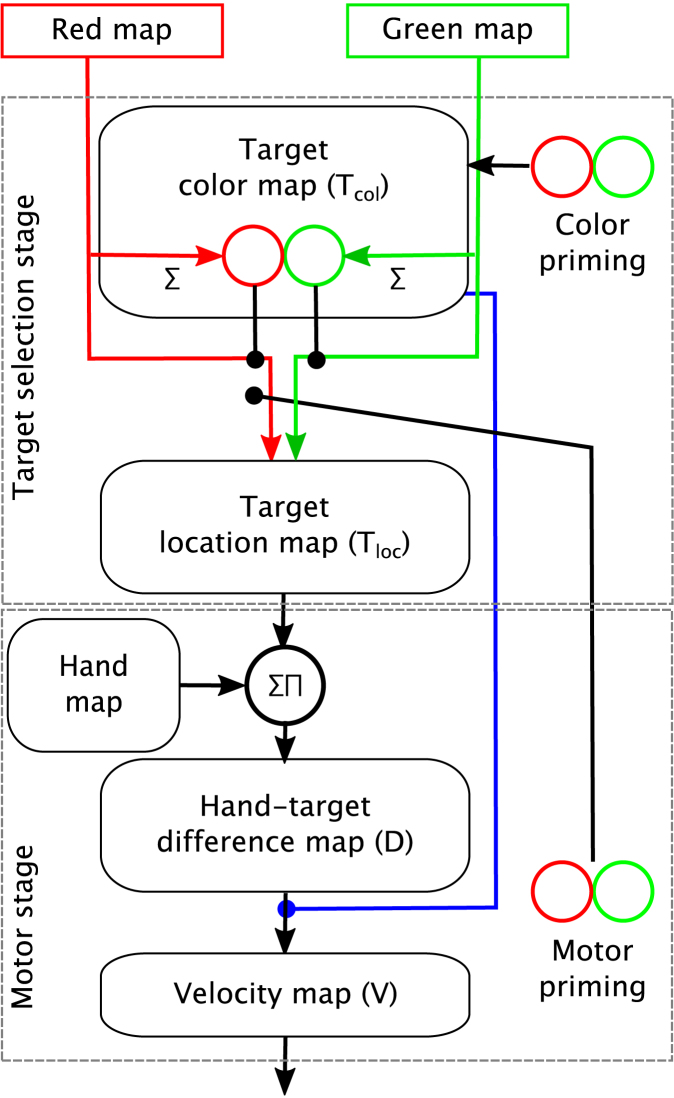
Overview of CoRLEGO’s architecture. The extensions present in the paper are the motor priming unit and the gating mechanism at the output of the D map controlled by a thresholded activation from target colour map (blue line). The dots in the graphics indicate modulatory connections. E.g. the target colour map weights the input of colour maps into the target location map (see main text for details). (For interpretation of the references to colour in this figure legend, the reader is referred to the web version of this article.)

The photographs show that we used a normal desk light (grey object next to the camera in Fig. 1) to keep the lighting roughly constant. For an easier detection of the robot arm blue markers were attached to the arm base and to the end effector. For the search items red and green coloured markers were used. These markers were detected with a combination of simple standard computer vision methods such as HSV-colour space, thresholding and erosion filters (see Strauss & Heinke, 2012 for details). At the beginning of each experiment the parameters of the methods had to be calibrated in order to adapt them to the lighting conditions. The output formed three two-dimensional binary maps feeding into the green map, red map and hand map (blue marker) in CoRLEGO’s model (see Fig. 2).

The output of CoRLEGO’s model is an activation blob which encodes a two-dimensional velocity vector in neural-field style (see next section for detailed description). To translate the neural-field encoding into speed commands for the LEGO motors, the distance of the centre of gravity of the activation blob to the centre of the V map was calculated. This Cartesian information was transformed into joint speeds following the standard approach of an approximation of the inverse of the Jacobian matrix.

### 2.2 Overview of CoRLEGO’s model

One of the inputs to the model are the red and green feature maps. The target selection stage consists of two maps, the target colour map and the target location map. The target colour map ($T_{\mathrm{col}}$ map, Fig. 2) detects the odd colour in the input image. The $T_{\mathrm{col}}$ map has two units representing the two colours, red and green. As input, each unit receives the total activation of the respective maps. A competition process then activates the unit with the larger input activation while the other unit’s activation is suppressed. Hence, this losing unit indicates the target colour (odd colour). The input to the target location map ($T_{\mathrm{loc}}$ map) is the topologically summed activation from the green colour map and the red colour map. The summation is weighted by the inverted output of the $T_{\mathrm{col}}$ map (black dots in Fig. 2; Eq. (A.5)). Hence, once the odd colour is determined by the target colour map only the respective colour map is fed into the target location map. Consequently competitive processes in the target location map activate units at the position of the odd-colour target. This output activation feeds into the motor stage. However note that the competition in both maps operates in parallel. Hence even before the odd-colour is determined the target location map begins building up a representation of all items in the display including the distractors. This simultaneous representation of items will be crucial for simulating curved trajectories as we will explain below.

In the motor stage the output of the target location map is transformed into a topological representation of the distance between end effector and target (hand-centred target map; D map). This representation is generated through a spatial correlation between the end effector map and the $T_{\mathrm{loc}}$ map (∑∏-symbol in Fig. 2). To understand this it is important to remember that both maps encode locations through activation blobs as set out by the DNF framework. The target location map has a blob at the location of the target (once the selection processes are completed) and the end effector map has a blob at the position of the end effector. Consequently a spatial correlation between these maps determines the spatial “distance” between the blobs (in the same way a temporal correlation between two Dirac pulses determines the time delay between the two pulses). The spatial correlation is implemented in a way that the origin of the effector-centred coordinates is aligned with the centre of the D map. Note that a biologically plausible implementation of the spatial correlation can be achieved with sigma–pi units. Sigma–pi units were first proposed by McClelland, Rumelhart, and Hinton (1986) (see Heinke & Humphreys, 2003 for another example of an application).

The output of the D map feeds into the velocity map (V map), the core of the motor stage. The velocity map encodes the arm’s velocity by using an activation blob. To be more specific, the blob’s x- and y-coordinates encode the arm’s velocity with respect to x- and y-directions. For instance a blob at the corner of a map corresponds to a maximal velocity in both directions while a blob in the centre of the map encodes zero velocity. Note that this velocity coding schema naturally aligns with the coding schema of the D map. A activation blob in centre of the D map encodes zero distance between target and endeffector. Furthermore, activation at the centre of the D map leads to an activation in the centre of the velocity map which in turn brings the arm to a halt. In other words, the encoding schema in the velocity map and the D map ensures that once the arm has reached the target the arm stops. It is also important to note that the activation in the velocity map changes in a moving blob style (Amari, 1977). The movement of the moving blob is directed by the target location via the D map. (This directing of the moving blob can be pictured as a ball, the moving blob, rolling on a planar surface whose slope can be modified.) As a consequence of the moving blob behaviour the arm changes direction and speed in a non-jerky human-like fashion.

During the course of a reach the moving blob behaviour plays out in the following way. At the beginning the blob is positioned at the centre of the velocity map (zero speed). Once the $T_{\mathrm{loc}}$ map begins to detect the target location the D map establishes the distance of the arm from the target and encodes it as an activation blob at the corresponding location. Consequently this blob in the D map directs the blob in the velocity map towards this position which encodes speed and movement directions in the velocity map resulting in a reach towards the target. However and importantly, competing distractors can also filter through to the velocity map which results in the distractors ‘pulling’ the moving blob in their direction allowing the model to simulate curved reaching trajectories found in the choice reaching tasks.

Finally, to model the POP-effect the model possesses “colour priming” units (see Fig. 2). These units influence the competition process in the target colour map by pre-activating the units within the map. If the model is set up to replicate the effect of target colour repetition the priming units pre-activate the colour unit corresponding to the odd-colour in the display. Thus, target selection on the whole is expedited and the influence of distractors is reduced, leading to shorter latencies and straighter trajectories. On the other hand, to simulate the switch of target colour at the end of a streak of repetitions the unit encoding the distractor colour is pre-activated and the influence of distractor is increased resulting in longer latencies and more curved trajectories. It is important to note that this architecture inherently links the two dependent measures, maximum deviation and initiation latency. This link is consistent with Song and Nakayama’s and Woodgate et al.’s (2015) findings in the pre-tDCS conditions. However (Woodgate et al., 2015) also found that this link is disrupted in the tDCS conditions. We will return to this important aspect when we develop the extension of CoRLEGO in the second part of this paper.

### 2.3 Results

Strauss and Heinke’s (2012) simulation results showed that CoRLEGO operates as expected. In particular, CoRLEGO is able to mimic Song and Nakayama’s (2006) POP-effect with the help of the colour priming neurons. In addition, the moving blob mechanism is able to generate the typical bell-shaped velocity profile of human reaches.

However it is important to note that CoRLEGO’s environment (see Fig. 1) is fairly noisy. The noise has several sources. For instance, the lighting conditions can change during reaches as well as from reach to reach. These variations results in the representations of the coloured squares in the colour maps to change in size thereby affecting the movement control. Note that the camera adds further to this noise. On the output side the mechanics of the LEGO arm and the motor servos add to the noise CoRLEGO has to deal with. In other words, this environment is reminiscent to the noise the brain has to deal with.

Interestingly, it turns out that for CoRLEGO to successfully execute the reaches in such a noisy environment the velocity map needs to be organized in a nonlinear fashion, meaning that the spatial resolution of the map changes with the speed the location encodes. To be more specific, at low velocities (the centre of the map) the map needs to have a fine-grained resolution, while at a high velocity, (the periphery of the map) the velocity resolution is coarser. This way the arm makes more precise movements when the end effector is close to the target (low velocity). Interestingly the structure of the map would match the structure of the retina and the visual cortex (e.g., Rovamo & Virsu, 1979), and is therefore a fairly plausible prediction. However, to date this prediction has not been tested.

### 2.4 Related brain areas

Since the aim of this paper is to provide a computational explanation for the results from our brain stimulation experiment, it is important to discuss how CoRLEGO can be related to the functionality of certain brain regions. The functions of the different maps in CoRLEGO can be related to assumed functions of brain areas. CoRLEGO’s colour maps are assumed to be related to V4 in the visual cortex (e.g. Zeki, 1984). The velocity map with its encoding of the movement speed can be seen as part of the motor cortex. The maps in the target selection stage with their attentional function are traditionally linked to the posterior parietal cortex (PPC). Similarly, the priming neurons could be located in the parietal cortex as activation in this region is correlated with the POP effect (see Kristjansson & Campana, 2010 for a review). However, it has recently become clear that the PPC is also involved in motor control (see Baldauf & Deubel, 2010 for a good review). In particular the parietal reach region (PRR) has been implicated in reach-related processing, e.g. coordinate transformations, similar to the one implemented in the D map (see Yttri, Wang, Liu, & Snyder, 2014 for most recent evidence).

## 3 Extension of CoRLEGO

In the earlier summary of Woodgate et al.’s (2015) findings we simply noted that the motor system may be involved in the guidance of attention when target properties are predictable. Even though these findings alone are interesting and novel, there are several intriguing details. In Section  3.1 we will present these details. In Section  3.2, to highlight the added value of CoRLEGO, we also discuss intuitively plausible explanations of the findings that can be ruled out in the framework of CoRLEGO. In Section  3.3, we present the extension of CoRLEGO we have chosen together with the simulation results.

### 3.1 Woodgate et al.’s (2015) findings

Woodgate et al. (2015) found that tDCS influences reaching movements only when the experimental design contain streaks of target colour repetitions (predictable target colour). To be more specific, anodal tDCS reduced the maximum deviation (MD) from the ideal movement path while cathodal tDCS increased the deviation. Note that this qualitative difference between anodal and cathodal effect is consistent with findings in movement experiments (e.g. Reis et al., 2009). However and importantly this effect was not found in the initiation latency (IL) (compare Fig. 3(c) and (b)). This effect also contrasts with Song and Nakayama’s and our findings that MD and IL are normally linked, i.e. an experimental manipulation that reduces IL also reduces MD. To the best of our knowledge, this is the first time such dissociation between IL and MD has been found. In this paper we will use CoRLEGO to explain this intriguing dissociation.

The data also contains other effects commonly found in similar behavioural studies such as individual differences in participants’ behaviour, practise effects, statistically non-significant experimental conditions, etc. As we were not interested in modelling these effects or conditions we reduced the data so that it constitutes a compact but veridical representation of the dissociation between IL and MD. The original study by Woodgate et al. (2015) first tested participants using Song and Nakayama’s (2006) CRT with up to five target colour repetitions without applying tDCS (pre-tDCS condition). Subsequently participants performed in the same task but this time either anodal or cathodal tDCS was applied. Finally they completed yet another block of this task without tDCS (post-tDCS condition). There was little difference between different lengths of streaks and the effect in the post-tDCS condition was the same across the streaks. Therefore we model only the experimental outcome from the fifth colour repetition in the three conditions, the pre-tDCS condition, the anodal post-tDCS condition and the cathodal post-tDCS condition. Moreover, Woodgate et al.’s (2015) findings with respect to a tDCS-effect in the switch condition were weaker than for the streak condition due to a lack of statistical power. Therefore the switch condition from the post-tDCS condition was not included. Note that this reduction is not only justifiable from a scientific standpoint but also from a technical one as simulations with CoRLEGO are time-consuming. And the LEGO arm does not provide enough space to generate statistically significant effects in maximum deviation for all these conditions. However we still wanted to demonstrate the ability of the extension of CoRLEGO to reproduce the difference between the switch and the streak condition. Therefore we included the switch condition from the pre-tDCS condition.

Finally it is important to recap that the anodal and cathodal tDCS conditions were conducted with different participants groups to avoid an interference effect between these two types of stimulation. Due to individual differences the two groups performed differently in their pre-tDCS condition. Therefore we averaged across these two experimental groups. Also these individual differences do not allow for a direct comparison of the post-tDCS conditions. We therefore determined the change in performance caused by tDCS for each group. The change in performance was then added to the averaged pre-tDCS performance. The resulting values were used to evaluate the model’s simulation results (see Fig. 3(b) and (c) for an illustration). Note that in order to simplify the terminology in the remaining part of the paper we simply refer to the averaged pre-tDCS conditions as the streak condition and the switch condition. To the tDCS effects corrected for individual differences we simply refer as the anodal tDCS condition and the cathodal tDCS condition.

### 3.2 Implausible solutions with CoRLEGO

The first obvious idea would be to assume that tDCS somehow influences the velocity map (e.g. its parameters) as it is assumed to be part of the motor cortex and the tDCS was applied to the motor cortex. This would be a parsimonious solution as it would not require the addition of more structures to CoRLEGO. However, any parameter change in the velocity map will affect the properties of the moving blob dynamic therefore affecting both dependent variables. In addition there is the problem that the tDCS effect depends on the predictability of the target colour. This perceptually-related information is not stored in the velocity map.

Interestingly an animal study by Zach, Inbar, Grinvald, Bergman, and Yaadia (2008) found that the motor cortex can form representations of colour information if the information is strongly related to movements (see Eisenberg, Shmuelof, Vaadia, & Zohary, 2011 for an fMRI-study with humans pointing in the same direction). Hence one can speculate that such representations are formed in the human brain when colours are highly movement-relevant (i.e. predictable of the position of movement target) and it is conceivable that our tDSC stimulation modulated these representations. These units, in turn, may feed into the colour priming units in the target selection stage (parietal cortex), i.e. supporting the priming effect. Such a pathway would be supported by well-known connections from the motor cortex to the parietal cortex. However, such a realization would not lead to a dissociation between IL and MD. Nevertheless, the possibility of perceptually related units in the motor cortex formed the basis for CoRLEGO’s extension as discussed in the following section.

### 3.3 Plausible implementation

The starting point of extending CoRLEGO was indeed priming neurons in the motor stage here termed motor priming units (see Fig. 2), as suggested in the previous section. However, rather than feeding them directly into the colour priming units they weight the influence of the colour target units at the input of the $T_{\mathrm{loc}}$ map (black line in Fig. 2; Eq. (A.6)). This weighting allows the modulation of the contrast between the two colour units. If the motor priming units are highly activated (anodal tDCS) the higher contrast between the two colour units lead to a short IL and straighter MD. In contrast, smaller activation of the motor priming units (cathodal tDCS) leads to less contrast consequently slower IL and more curvature. In other words, the motor priming units influence IL and MD similarly to the colour priming. Nevertheless the separate pathways allow us to implement an dissociation between MD and IL. We introduced a gating mechanism at the output of the $T_{\mathrm{loc}}$ map that only passes on output activation if the level of the $T_{\mathrm{col}}$ map surpasses a set threshold (see Eq. (A.7)). Thus the moving blob in the velocity map is only pushed off the centre once the threshold is passed. This mechanism ensures that IL only depends on perceptual priming and is little affected by the motor priming units (tDCS).

However, extensive tests of the gating mechanism at the output of the $T_{\mathrm{loc}}$ map under the noisy conditions of CoRLEGO did not lead to stable results (see Section  3.5 for an explanation of how the parameters were fitted). This was mainly due to the characteristics of how the priming (pre-activation) influences the build-up of activation in the dynamic neural fields. The longer the build-up the larger the activation differences between different levels of pre-activation. Hence to achieve a good priming effect in noisy conditions it is important to place the threshold (of the gating mechanism) relatively high. However such a delay in passing the threshold also leads to the completion of the target selection therefore resulting in little influence of distractors on the reaching trajectory. Therefore, we decided to apply the gating mechanism at the output of the D map (see blue line in Fig. 2), so that the D map acts as short-term storage for the distractor activation. This way the threshold can be fairly high, but because the D map (CoRLEGO’s PRR) is slow to follow changes at the target location map the distractor can still can produce trajectory deviation. Interestingly, the design decision is also supported by very recent experimental evidence on the PRR (Yttri et al., 2014), where lesioning the PRR affects the onset of reaching but not the selection of movement targets (attention) or the control of movements as such.

### 3.4 Simulation method, data analysis and evaluation of model

The size of the target objects was 3.5 cm × 3.5 cm. Targets were located on a virtual circle with the radius of 22 cm at 45°, 90°, and 135° (from left to right). The centre of this circle was the starting position of the robot arm’s hand. The starting position was located 9 cm in front of the arm’s base (shoulder). The odd-colour target object was placed on the right side. In each of the four conditions (switch, streak, anodal and cathodal tDCS) we conducted five trials.

For the purpose of data analysis the raw data of each trajectory was pre-processed with the following steps. First, a B-Spline (3rd order) of the raw data points was calculated which reduced noise and normalized the trajectories to 100 data points. Second, we applied a Butterworth filter (2nd order) with a cut-off frequency of 1/20 of the sampling rate of the camera in order to reduce the noise even more.

The maximum deviation (MD) was calculated by dividing the maximum deviation of the data points from a straight line by the length of this line. The straight line was determined by the start and end points of the trajectory. The initiation latency (IL) was defined as the point in time when the velocity was higher than 0.5 cm/s for the first time following the start of the simulation. After starting a trial the position of the end-effector was recorded until the target was reached. The arm was considered to have reached the target when the encoded velocity in the V map fell under a threshold value of approximately 0.5 cm/s.

The most obvious way of evaluating models is by comparing the quantitative differences between model and data, e.g. using sum of squared difference. For obvious reasons such a simple quantitative comparison is not adequate in context of CoRLEGO. Instead we focused on a qualitative assessment of CoRLEGO and examined whether the response pattern was qualitatively the same as human. For instance, CoRLEGO was expected to show no significant effect for IL from tDCS while should be a significant effect MD. These effects were analysed by using t-tests. It is worth noting that such a qualitative evaluation is seen as legitimate approach in the literature (see Heinke, 2009, Pitt et al., 2006, Wills and Pothos, 2012 for more discussions). Furthermore, even though such a qualitative evaluation seems sufficient to us we also included an evaluation that assessed the relative magnitudes between conditions. Therefore we re-scaled CoRLEGO’s responses with the ratio between the overall means of human responses and CoRLEGO’s responses. This minimizes overall differences between human data and CoRLEGO while at the same time keeps relative differences between conditions.

### 3.5 Results

Before a test began the parameters of the image processing were adapted to the present lighting conditions. The parameters of the DNFs used very similar parameters as in Strauss and Heinke (2012) (see Appendix A, Appendix B for details). These parameters are crucial for securing a selection of the target and a successful control of the reaching movement. For achieving the target behaviour only three parameters are crucial: the strength of the perceptual priming, the threshold of the gating mechanism and the weighting of the colour maps (motor priming) (see Appendix A, Appendix B for details). The parameters were modified manually until we found a good approximation of the target behaviour particularly in terms of its qualitative pattern. The parameter fitting is not very complex as it turned out that the new architecture enables the user to establish good parameter values relatively independently. The simplicity of this process is important as the simulations with CoRLEGO are fairly time-consuming.

The parameter fitting procedure followed three steps. First a relatively high level of pre-activation of a colour unit (perceptual priming) had to ensure a fairly large difference between streak and switch condition in both MD and IL. But the level of pre-activation cannot be too high as otherwise the wrong colour would be selected in the switch condition. Also the streak condition needs to leave enough room for improvement in the MD-effect of the anodal tDCS condition. Second the threshold of the gating mechanism needs to be high enough so that there is a significant priming effect (see Section  3.3 for an explanation). But it cannot be set too high as an overly delayed IL would lead to a complete suppression of the distractors therefore exhibiting no curved trajectories. During these first two steps the weighting of the colour maps (motor priming) was set to a middle range value. This way the third step allowed an increase and decrease of this factor to modify MD for both the anodal and cathodal conditions. But the decrease needs to be limited so that the target colour map still has an influence on the target location map.

Fig. 3 and Table 1 show the results of the parameter fitting. The pairwise comparisons in Table 1 demonstrate that our model successfully mimics the pattern of the experimental outcome. A quantitative comparison between data and simulation in Fig. 3(b) and (c) indicates some minor differences. In particular the priming parameter for the switch condition led to higher switch effect on maximum deviation than in Woodgate et al.’s (2015) data. However, Woodgate et al.’s (2015) data showed an unusually small switching effect compared to other experiments we conducted with the same procedure. This diminished effect may have been due to the tDCS apparatus distracting participants.

## 4 Discussion

We presented an extension of a neurobiologically inspired robotics model (CoRLEGO). CoRLEGO models leakage of attentional selection into the motor system. The extension aimed to explain our findings that tDCS over the motor cortex modulates the colour priming effect (Woodgate et al., 2015). In particular, the extension aimed to explain why tDCS affected the maximum deviation of reaching movements and not the initiation latency. The new CoRLEGO suggests that colour priming based on the perceptual system is reflected in the initiation latency whereas the maximum deviation is affected by both perceptual- and motor-based priming. Furthermore, the detection of the odd colour leads to CoRLEGO initiating the movement (gating mechanism). In other words, the movement tends to begin once a task-relevant global property of the search display was determined. CoRLEGO’s motor priming neurons support the spatial selection of the movement target via the feedback from motor cortex to the visual system. Hence in a more general sense, the purpose of the feedback connections is to enhance the detection of movement relevant information, i.e. the spatial location of the odd-colour target (as opposed to selecting information that is not directly relevant to the movement, i.e. the colour itself). Interestingly CoRLEGO’s usage of feedback connections is consistent with Baldauf and Deubel’s (2010) theory of visual preparation. Their theory suggests that the feedback connections guide visual attention to extract movement-relevant information from visual stimuli.

There are other predictions that can be derived from CoRLEGO. The moving blob activation in the velocity map should be found in animal studies or fMRI studies with high resolution. Interestingly such a prediction is feasible as the moving blob behaviour is similar to the travelling waves found in a variety of brain areas including the motor cortex (e.g. Takahashi, Saleh, Penn, & Hatsopoulos, 2011). In particular a recent animal study by Riehle, Wirtssohn, Grün, and Brochier (2013) found that activation travels from arm-related areas to hand-related areas during reach-to-grasp movements. In other words, the shift of activation mirrors the different phases of the movement. Hence CoRLEGO’s moving blob behaviour suggests that these shifts also encode movement parameters. Finally the odd-colour detection threshold in the model’s PRR also predicts results for an fMRI study. Here the activation flow, indexed with dynamic causal modelling or event-related fMRI, should be small prior to movement onset before increasing at the point of initiation (bar some signal latencies).

On a wider perspective CoRLEGO falls into the category of closed-loop control models of human movements. This type of model contrasts with open-loop control models. In these models the arm movement is planned, e.g. in terms of the sequence of muscles activations, prior to its execution. For a long time, this type of model dominated the explanations of human movements. However recent evidence and computational modelling favour the closed-loop approaches (see Todorov, 2004 for a review). But then again, this evidence is mainly based on experiments involving single targets. In multi-object situations such as the one discussed in this paper it is possible that the brain adopts a different strategy whereby the movements to different target positions are pre-planned and “averaged” trajectories are executed before the final target position is known to the motor system. In fact this interpretation has been put forward by Flanagan and colleagues (e.g. Stewart, Baugh, Gallivan, & Flanagan, 2013). In future work a computational model of this theory will have to be contrasted with CoRLEGO’s closed-loop approach.

In the introduction of this paper we noted that choice reaching tasks have been used to tap into other cognitive processes such as language processing (Spivey et al., 2005) or representations of numbers (Song & Nakayama, 2008b). For instance, Song and Nakayama (2008b) presented single digits on the centre of a screen and two boxes on either side of the digit. Participants were asked to reach for the box on the left side if the digit was smaller than five and if larger than five to the right side. Song and Nakayama (2008a) were able to show that the trajectories were more curved towards the centre of the screen the closer the digit was to five, thus pointing towards a spatial representation of numbers. Of course CoRLEGO would have to be extended and such spatial number representation introduced. However, we also expect that the central mechanism in CorLEGO, the moving blob mechanism, would be able to mediate the selection process in this spatial representation of numbers into the reaching movement. Similarly (Spivey et al., 2005) presented two pictures on either side of the screen and asked the participants to move a mouse pointer towards the picture with a dark frame. If the two pictures were phonologically similar (e.g. picture and pickle) the movement took a more central line whereas if the two pictures were phonologically dissimilar (e.g. picture and jacket) movement more biased towards the target image. Again a new version of CoRLEGO could include such phonological similarities, while the moving blob mechanism should be able to pass on these characteristics of the selection process. These discussions of future work highlight the potential of CoRLEGO’s moving blob mechanism for modelling other leakage effects.

## 5 Lessons for neurobiologically inspired robotics

The work has several lessons for neurobiologically inspired robotics. Even though the set-up greatly simplified the visual environment and the complexity of movement control humans usually have to deal with, the set-up still contained enough noise to require the designer to rethink the architecture. As a result it was necessary to change the spatial organization of the velocity map and the influence of the perceptual system on the motor cortex. This experience demonstrates the importance of building a robot; in a possible simulation environment the designer would be more tempted to simply adjust the environment. It is also worth noting that the whole architecture including the dynamic neural fields must be robust to noise giving additional credence to the model.

One caveat of this approach is that running simulations can be time-consuming, as they are with CoRLEGO. Hence, extensive parameter optimization is not really possible in this framework. Similarly extensive model comparisons as suggested in the outlook section of the discussion are not feasible. In these applications it would be necessary to set up simulation of the robot’s environment and any parameter optimization or model comparison can be done in the simulation environment. However, the final test for the success of the approach has to be made in a real-world scenario, as it is unlikely that the simulations can capture all characteristics of a real-world scenario. In other words, a combination of simulation and real-world scenario presents a way forward in neurobiologically inspired robotics.

Another perhaps more novel aspect of our robotics-based approach is the usage of a simple, cheap LEGO arm rather than an expensive industrial robot arm. Our arm is also very easy to control with its two degrees of freedom and is custom made to capture the crucial characteristics of experimental findings. In other words, LEGO allows the researcher to build a customized simple robot in order to answer a very specific scientific question. In contrast, an industrial robot arm would have a lot of baggage which the designer would need to deal with, but which are not relevant to the scientific question. Moreover the usage of LEGO will allow the complexity of the arm to increase (e.g. adding a gripper) while building on the present results. Nevertheless, the lessons learnt from the work with a LEGO robot should be transferable to an industrial robot.

CoRLEGO highlights yet again the power of maps in general and in particular the topological representation of movement parameters. It demonstrates that this framework can establish an elegant link between perceptual processing and movement control. However, it also indicates that the dynamic within the two systems may be very different, i.e. the perceptual system exhibits a decision style of dynamics while the motor system shows smooth transitions between states. Moreover the maps design an efficient and faster method to control movements as both systems can operate in parallel.

Finally, in the context of a technical solution it is not clear why it is necessary that the motor system needs to contribute to the detection of movement relevant information as suggested by CoRLEGO. It is possible that this solution is somehow owed to constraints imposed by the neurological substrate (e.g. speed signal transmission) and possibly not important for technical solutions. This is however a question for future research.

## Figures and Tables

**Table:** 

Map	hue	Δhue	sv	ero
${\mathrm{col}}_{\mathrm{blue}}$	230	50	20	1
${\mathrm{col}}_{\mathrm{red}}$	4	18	35	2
${\mathrm{col}}_{\mathrm{green}}$	110	50	20	2

**Table:** 

Map	τ	β	h	$g_{\mathrm{inh}}$	$c_{\mathrm{exc}}$	$\sigma_{\mathrm{exc}}$	$c_{\mathrm{inh}}$	$\sigma_{\mathrm{inh}}$	$c_{q}$	$\sigma_{q}$
B	25	12	−2	0.3	80	3	20	10	0.05	10
Hand map	2	12	−0.5	0.2	20	3	0	1	0.05	1
$T_{\mathrm{col}}$	1500	12	−1	7	5	0.1	10	0.1	0.05	1
$T_{\mathrm{loc}}$	150	1.5	−0.5	0.3	35	4	0	8	0.05	1
D	15	12	−0.5	0.1	10	3	0	3	0.05	1
V	24	2	−1	0.4	20	5	0	1	0.05	5

**Table:** 

Condition	Cathodal tDCS	Anodal tDCS	Streak	Switch
p	0.6	1	0.75	0.75

**Fig. 3(a):**
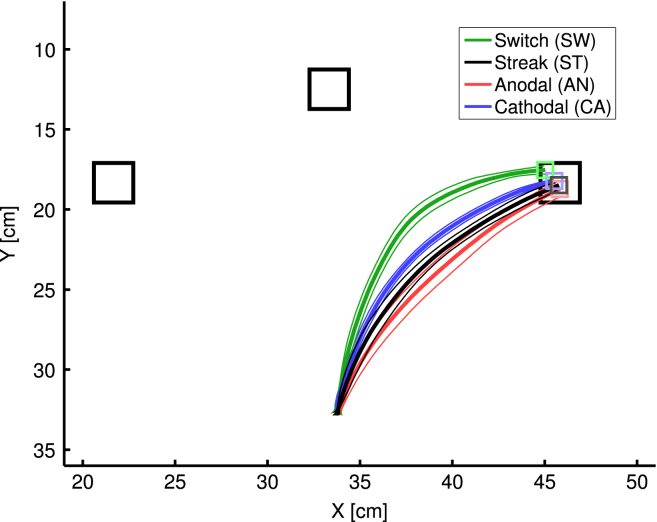
(a) Averaged trajectories. The thin lines indicate the standard error from five trials in each condition.

**Fig. 3(b):**
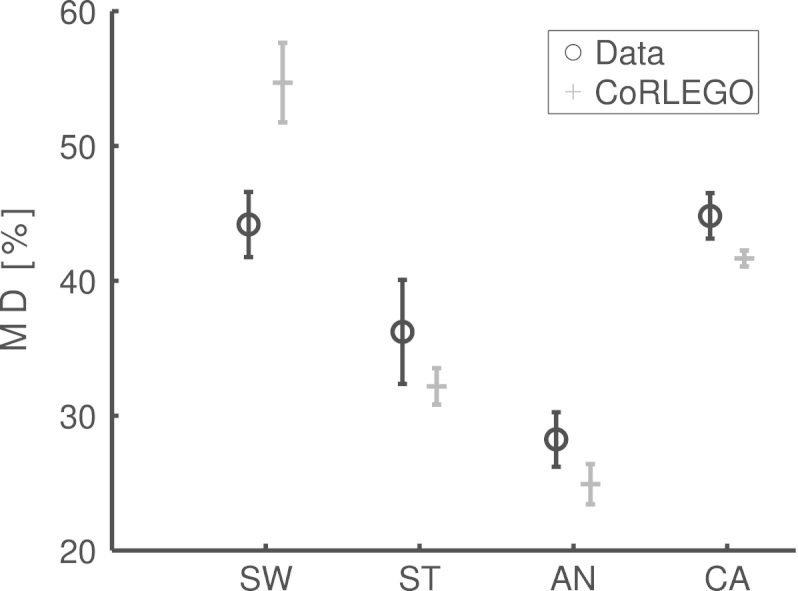
(b) Maximum deviations (MD).

**Fig. 3(c):**
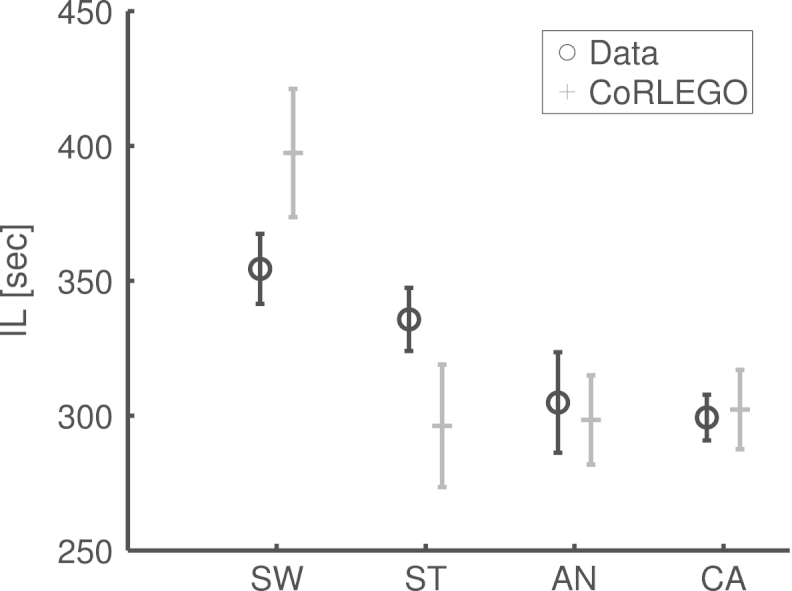
(c) Initiation latencies (IL).

**Table 1:** t-tests on CoRLEGO’s results. The tests demonstrate that CoRLEGO was able to replicate the behavioural pattern found by Woodgate et al. (2015). The bottom lines show the results for initiation latency (IL) and the top lines the results for the maximum deviation (MD). The results in bold indicate a significant comparison at an α-level of 0.05. The abbreviations stand for the four conditions: switch (SW), streak (ST), anodal tDCS (AN) and cathodal tDCS (CA) conditions.

	SW	AN	CA
ST	t=3.07; p=0.015	t=0.08; p=0.94	t=0.83; p=0.22
	t=6.95; p<0.01	t=3.62; p=0.1	t=6.44; p<0.01
AN	t=3.42; p=0.01	–	t=−0.18; p=0.87
	t=9.01; p<0.01	–	t=−10.48; p<0.01
CA	t=3.40; p=0.01	–	–
	t=4.33; p<0.01	–	–

## Appendix A Mathematics of the extension of CoRLEGO

This section focuses on the mathematical description of the extension of CoRLEGO. Further details on the implementation of CoRLEGO can be found in Strauss and Heinke (2012). The maps involved in the extension of CoRLEGO are the target colour map ($T_{\mathrm{col}}$), the target location map ($T_{\mathrm{loc}}$), hand-target difference map (D), the velocity map (V), the green map (${\mathrm{col}}_{\mathrm{green}}$) and the red map (${\mathrm{col}}_{\mathrm{red}}$). To set the scene for the extensions, we also present the equations for CoRLEGO’s dynamic neural fields.

### A.1 Dynamic neural field

Our implementation follows Faubel and Schoener’s (2008) approach which turned out to be a more robust than the original implementation by Amari (1977).

(A.1) $$\tau \overset{˙}{u}\left(x,t\right)=-u\left(x,t\right)+h+s\left(x,t\right)+{\mathrm{exc}}_{\mathrm{loc}}\left(x,t\right)-{\mathrm{inh}}_{\mathrm{loc}}\left(x,t\right)-{\mathrm{inh}}_{\mathrm{glob}}\left(x,t\right)+q\left(x,t\right)\text{.}$$

Hereby, τ is a time parameter which defines how fast the DNF adapts towards changing inputs, u(x,t) stands for the field activation at time t and location x, h<0 is the resting level of the field, s(x,t) is the external input of the field, w(x) is the activation kernel function and q(x,t) is normally distributed Gaussian noise. Note that x is two-dimensional for the most DNFs in my model. Thus, ${\mathrm{exc}}_{\mathrm{loc}}$ and ${\mathrm{inh}}_{\mathrm{loc}}$ define the local excitation and inhibition with the following equation:

(A.2) $${\mathrm{exc}}_{\mathrm{loc}}\left(x,t\right)=\underset{x^{\prime }}{\sum }w_{\mathrm{exc}}\left(x-x^{\prime }\right)f\left(u\left(x^{\prime },t\right)\right)\text{.}$$

Next, the global inhibition ${\mathrm{inh}}_{\mathrm{glob}}$ depends on the summarized field activation and the parameter $g_{\mathrm{inh}}$:

(A.3) $${\mathrm{inh}}_{\mathrm{glob}}\left(x,t\right)=g_{\mathrm{inh}}\underset{x^{\prime }}{\sum }f\left(u\left(x^{\prime },t\right)\right)\text{.}$$

Finally, the kernel $w_{k}$ with its parameters $\sigma_{k}$ (kernel width) and $c_{k}$ (kernel strength) is defined with the following equation:

(A.4) $$w_{k}\left(x\right)=\frac{c_{k}}{\sigma_{k}\sqrt{2\pi }}\exp\left(-\frac{{\left|x\right|}^{2}}{2\sigma_{k}^{2}}\right)\text{.}$$

Kernel functions define how connections spread out through the DNF and how large the radius of influence of a neuron in its neighbourhood is. They are applied in all DNFs in the local excitation, inhibition and the noise term q.

### A.2 Input to the target location map

In CoRLEGO’s original version the input to the target location map ($T_{\mathrm{loc}}$ map) is the topologically summed activation from the green colour map and the red colour map. The summation is weighted by the inverted output of the $T_{\mathrm{col}}$ map:

(A.5) $$s_{T_{\mathrm{loc}}}\left(x,t\right)=\left(1-f_{T_{\mathrm{col}}}\left(u_{T_{\mathrm{col}}}\left(\mathrm{green},t\right)\right)\right)\cdot {\mathrm{col}}_{\mathrm{green}}\left(x,t\right)+\left(1-f_{T_{\mathrm{col}}}\left(u_{T_{\mathrm{col}}}\left(\mathrm{red},t\right)\right)\right)\cdot {\mathrm{col}}_{\mathrm{red}}\left(x,t\right)\text{.}$$

To include the influence of motor priming (tDCS stimulation) the influence of the colour neurons is modulated with a factor (p):

(A.6) $$s_{T_{\mathrm{loc}}}\left(x,t\right)=\left(1-p\cdot f_{T_{\mathrm{col}}}\left(u_{T_{\mathrm{col}}}\left(\mathrm{green},t\right)\right)\right)\cdot {\mathrm{col}}_{\mathrm{green}}\left(x,t\right)+\left(1-p\cdot f_{T_{\mathrm{col}}}\left(u_{T_{\mathrm{col}}}\left(\mathrm{red},t\right)\right)\right)\cdot {\mathrm{col}}_{\mathrm{red}}\left(x,t\right)\text{.}$$

### A.3 Gating mechanism

The extension of CoRLEGO included a gating mechanism of the D map input ($V_{D}$) into the velocity map:

(A.7) $$V_{D}\left(x,t\right)=\left(g\left(f_{T_{\mathrm{col}}}\left(u_{T_{\mathrm{col}}}\left(\mathrm{green},t\right)\right)\right)+g\left(f_{T_{\mathrm{col}}}\left(u_{T_{\mathrm{col}}}\left(\mathrm{red},t\right)\right)\right)\right)\cdot f_{D}\left(u_{D}\left(x^{\prime },t\right)\right)$$

g(⋅) is a simple threshold function with the parameter Θ as threshold. If one of the two colour units in the colour target map ($T_{\mathrm{col}}$) passes this threshold the activation output of the D map is passed onto the velocity map.

## Appendix B Parameters

### B.3 Critical parameters for simulating data

The threshold (θ) in the gating mechanism was set to 0.3. The factor p in the input to the location map was set to different depending on the condition CoRLEGO was set up to simulate:

Note that the differing behaviour between the streak- and the switch-condition results from priming the same colour as the odd-colour in display (streak-condition) or priming the other colour as the odd-colour in display (switch-condition).

The priming (pre-activation) of the target colour map was implemented by setting the input of the to-be-primed colour units to 4 and then simulated the target colour map without any other map or input. This simulation continued until the map reached stable state. Only after this pre-activation phase was completed the whole of CoRLEGO resumed normal operations and the colour units of the target colour map received the total activation of the respective colour map.
